# Evaluation of edible coating on quality, biochemical components and *microbial* community under cooling and freezing conditions on fresh-cut artichoke

**DOI:** 10.1038/s41598-026-63629-2

**Published:** 2026-08-08

**Authors:** Amira A. AboElmakarem, Sabah Sh A. El-Sayed

**Affiliations:** 1 Postharvest and handling Vegetables Research Department, Agriculture Research Centre, Horticulture Research Institute, Giza, Egypt; 2 Food Engineering and Packaging Department, Agriculture Research Centre, Food Technology Research Institute, Giza, Egypt

**Keywords:** Fresh-cut artichoke, Biopolymer coatings, Essential oil enrichment, Individual quick freezing, Oxidative stability, Microbial quality, Postharvest preservation, Functional food, Biochemistry, Biotechnology, Chemistry, Microbiology

## Abstract

This study evaluated the effectiveness of edible coatings combined with natural essential oils in improving the postharvest quality, nutritional value, and microbial stability of artichokes under different storage conditions. The experiment was conducted over two growing seasons (2024–2025) using a French hybrid cultivar. Artichokes were coated with whey protein, gum Arabic (GA), or carboxymethyl cellulose (CMC), and stored under refrigeration (0 °C), individual quick freezing (IQF) (-30 °C), and combined freezing–thawing conditions in polyethylene and polypropylene packaging. Results showed that all edible coatings significantly enhanced physicochemical and microbiological quality compared with uncoated controls. Coated samples exhibited reduced weight loss, respiration rate, and ethylene production, along with improved retention of key nutrients, including carbohydrates, sugars, protein, folic acid, inulin, cynarin, phenolics, and ascorbic acid. GA and whey protein coatings demonstrated the most pronounced effectiveness. Microbial analyses revealed substantial reductions in total bacterial count, yeasts, and molds in coated samples, consistent with the antimicrobial activity of essential oil enriched formulations. Additionally, coatings reduced oxidative stress indicators, including malondialdehyde, hydrogen peroxide, and superoxide anion levels, while suppressing polyphenol oxidase activity. Overall, edible coatings enriched with natural essential oils effectively preserved quality and extended the shelf life of artichokes by limiting physiological deterioration, microbial growth, and oxidative damage. These results highlight their potential as sustainable postharvest preservation strategies for fresh-cut horticultural products.

**Supplementary Information:**

The online version contains supplementary material available at 10.1038/s41598-026-63629-2.

## Introduction

Artichoke (*Cynara cardunculus* var. *scolymus*) is a perennial plant belonging to the Asteraceae family and is widely recognized for its high nutritional and functional value. It is rich in dietary fiber, vitamins, minerals, and antioxidant compounds, making it an important functional food with increasing global demand. Due to its high content of bioactive constituents, artichoke has been extensively studied for its health-promoting properties, particularly its antioxidant and metabolic regulatory effects^1,2^.

Artichoke shows marked varietal diversity across agro-climatic regions, reflecting long-term cultivation and adaptation. It is primarily produced in the Mediterranean basin, with Italy, Spain, France, Egypt, and the United States (California) as leading producers under favorable climatic conditions. The globe artichoke is the dominant cultivated type, characterized by large edible flower heads and wide commercial relevance. Considerable regional variation exists within cultivated germplasm, including green and violet types differing in head morphology, tenderness, and sensory quality. French and Italian cultivars are particularly distinguished by differences in head shape and pigmentation, and are used for both fresh consumption and processing. In Spain, ‘Blanca de Tudela’ represents a key local cultivar valued for quality and adaptability. Modern breeding efforts have generated hybrid and thornless cultivars with improved yield stability, uniformity, and resistance to abiotic stress and postharvest losses. Egypt plays a significant role in global production, benefiting from favorable growing conditions and early export windows^3,4^. Overall, the diversity of artichoke cultivars underscores its strong agronomic adaptability and commercial importance as a functional vegetable crop.

The hepatoprotective activity of artichoke is mainly associated with its phenolic constituents, particularly cynarin, chlorogenic acid, and flavonoids. These compounds reduce oxidative stress by scavenging reactive oxygen species and protecting hepatocytes from lipid peroxidation. In addition, they enhance bile secretion, which facilitates detoxification processes and improves lipid metabolism. The anti-inflammatory effects of these bioactive molecules further contribute to liver protection by reducing hepatic inflammation and supporting overall liver function^1,2^.

Artichoke also exhibits significant antidiabetic potential due to its content of inulin, chlorogenic acid, and flavonoids. Inulin, as a soluble dietary fiber, delays glucose absorption and helps regulate postprandial blood glucose levels. Chlorogenic acid contributes to glucose homeostasis by inhibiting glucose-6-phosphatase activity, thereby reducing hepatic glucose output. Furthermore, flavonoids enhance insulin sensitivity and reduce oxidative stress, which plays a key role in the development of diabetic complications. These combined mechanisms highlight the potential of artichoke as a functional food in glycemic control^5^.

Despite its nutritional and medicinal importance, artichoke is highly perishable after harvest due to rapid physiological deterioration, including moisture loss, enzymatic browning, wilting, and microbial spoilage. These processes significantly reduce shelf life and marketability, especially during storage and transportation.

Biopolymer-based edible coatings have emerged as an effective and sustainable postharvest preservation strategy by forming semi-permeable films that regulate gas exchange, reduce moisture loss, and delay physiological deterioration in fresh produce. Key biopolymers such as whey protein, GA, and CMC provide complementary functional properties that enhance coating performance. Whey protein offers excellent film-forming ability, mechanical strength, and oxygen barrier properties due to its globular protein structure. (GA) (Talha gum), a complex polysaccharide–protein exudate from *Acacia seyal* and *Acacia senegal*, contributes superior emulsifying, stabilizing, and film-forming characteristics owing to its arabinogalactan-rich structure which account for approximately 85–95% of its structure, along with a minor protein fraction (about 1–3%) that forms arabinogalactan–protein complexes responsible for its excellent emulsifying properties. In addition, it contains glycoproteins, oligosaccharides, and mineral constituents such as calcium, potassium, and magnesium. The polysaccharide backbone is primarily composed of arabinose, galactose, rhamnose, and glucuronic acid residues, which contribute to its high solubility, viscosity, and film-forming ability^6^. CMC, a water-soluble cellulose derivative, improves coating uniformity, flexibility, and moisture barrier efficiency, thereby enhancing structural integrity of the coating matrix.

The functional efficiency of these biopolymer matrices can be further enhanced through the incorporation of essential oils, such as lemon, peppermint, and clove oils, which act as natural bioactive additives. These essential oils are rich in volatile compounds including Lemonene, menthol, and eugenol, which exhibit strong antimicrobial, antioxidant, and antifungal activities. Their antimicrobial action is mainly attributed to the disruption of microbial cell membranes and leakage of intracellular components, while their antioxidant properties help in reducing lipid peroxidation and oxidative stress in plant tissues. When incorporated into biopolymer-based coatings, essential oils are gradually released from the polymeric matrix, providing sustained antimicrobial protection and improving the overall functional performance of the coating system. This synergistic interaction between biopolymers and essential oils enhances barrier properties, suppresses microbial growth, reduces respiration rate, and delays senescence, ultimately leading to improved postharvest quality and extended shelf life of fresh produce during storage^7–9^.

In addition to coating technologies, advanced storage methods such as refrigeration (0 °C, 95–98% RH) and (IQF) play a crucial role in preserving postharvest quality by slowing metabolic activity and minimizing structural damage^10^.

However, limited studies have investigated the combined effect of biopolymer-based edible coatings and advanced storage technologies on artichoke quality preservation. In particular, the synergistic interaction between coating formulations and storage conditions remains insufficiently explored.

Therefore, the present study aims to evaluate the effect of edible coatings (whey protein, GA, and CMC) enriched with essential oils in combination with two storage strategies (refrigeration and IQF) on the physicochemical, microbial, sensory, and mechanical properties of artichokes during storage.

The novelty of this work lies in providing an integrated preservation approach that combines natural biopolymer coatings with advanced storage technologies to enhance shelf life and maintain quality attributes of artichoke under postharvest conditions.

**Fig. 1:**
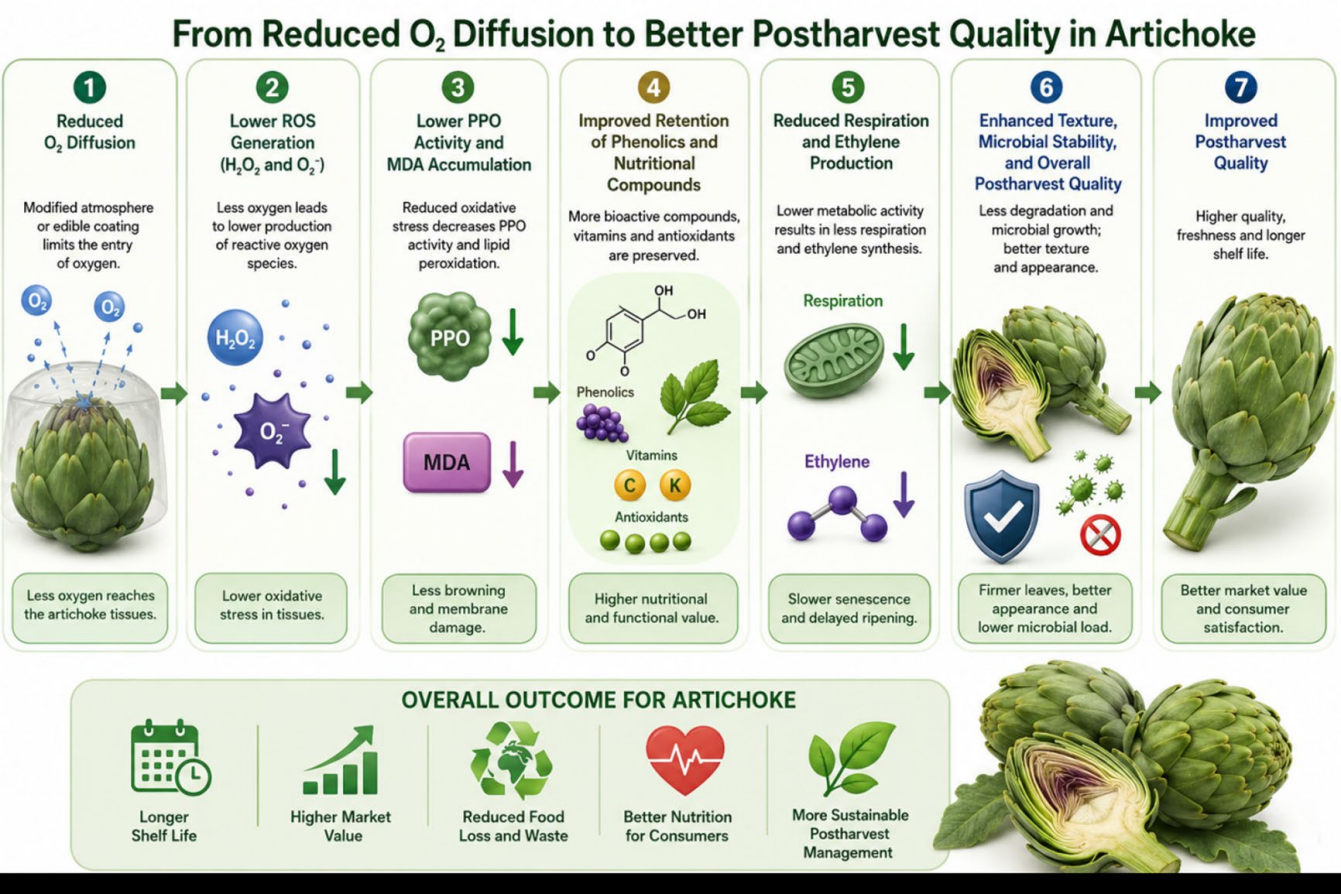
Postharvest preservation mechanism and quality enhancement pathways of active coatings on artichokes.

We hypothesize that the synergistic application of edible coatings and low-temperature storage will reduce oxidative stress, inhibit microbial growth, and improve retention of bioactive compounds in artichoke during storage (Fig. 1).

## Materials and methods

### Plant material and experimental design

The artichoke experiment was conducted over two consecutive growing seasons (2024–2025). A French hybrid cultivar was used and obtained immediately after harvest from Rayyan Farm and Factory Fresh, Kafr El-Dawar, Beheira Governorate, Egypt. To minimize enzymatic browning and oxidative deterioration, the harvested artichokes were immersed in a 2% (w/v) ascorbic acid solution prior to further processing to preserve it until it is transported to the laboratory.

### Sample preparation and packaging

Following pretreatment, artichoke samples were coated with edible coatings using a brush application technique and subsequently air-dried at ambient temperature to ensure uniform film formation. The coated samples were packaged in low-density polyethylene (LDPE) bags (47 × 33 cm; 0.03 mm thickness) and polypropylene (PP) bags (40 μm thickness), supplied by the Department of Manufacturing, Packaging, and Food Packaging Engineering Research, Food Technology Institute, Agricultural Research Center, Giza, Egypt. All samples were stored for further analyses.

### Preparation of edible coating formulations

#### Whey protein coating

Whey protein was obtained from (≥ 95% purity, Sigma-Aldrich, St. Louis, MO, USA) according to the manufacturer’s specifications. The whey protein used without additional flavoring agents, sweeteners, or other commercial additives. A solution was prepared by dissolving 100 g of whey protein in 1 L of distilled water. The solution was heated at 45 °C for 30 min under continuous stirring. A calcium chloride (CaCl₂) solution (20 g L⁻¹) was prepared separately and heated at 45 °C for 15 min under stirring. The two solutions were then combined under continuous stirring. Subsequently, 20 mL of lemon oil and 2 mL of Tween 20 (20%) were added as an emulsifier. The final coating was applied to the samples using a brush technique. All treatments were performed at room temperature (20–25 °C). After coating, samples were air-dried under ambient conditions. This treatment was applied to enhance tissue firmness and reduce enzymatic activity during storage^11^.

Calcium chloride (CaCl₂) was incorporated into the coating formulations because calcium ions play an important role in maintaining cell wall integrity through the formation of calcium pectate cross-links within the middle lamella. This interaction enhances tissue firmness, delays softening, reduces membrane permeability, and contributes to the suppression of enzymatic browning and senescence-related metabolic activities during storage. In addition, calcium treatments have been widely reported to improve postharvest quality and extend the storage life of fresh fruits and vegetables^12,13^.

#### Gum Arabic (GA) coating (*Acacia seyal*)

GA (Talha gum) derived from *Acacia seyal* was obtained from a certified local supplier. The material was cleaned, finely ground, and used without preservatives or additives.

A solution was prepared by dissolving 100 g L⁻¹ GA in distilled water, followed by heating at 60 °C for 30 min under continuous stirring until complete dissolution. The solution was cooled to room temperature before the addition of 20 mL peppermint oil and 2% Tween 20 as an emulsifier. The coating was applied using a brush technique. GA served as the sole film-forming biopolymer and was used to enhance moisture retention and postharvest quality^14^.

#### Carboxymethyl cellulose (CMC) coating

CMC were obtained from (Sigma-Aldrich, St. Louis, MO, USA) was used without further purification. A solution was prepared by dissolving 60 g of CMC in 750 mL of distilled water under stirring, followed by the addition of 250 mL ethanol. The mixture was heated to 80 °C until complete dissolution. Finally, 20 mL clove oil and 2% Tween 20 were added. The coating was applied using a brush technique. The formulation was prepared freshly prior to use^15^.

#### Control: Uncoated samples served as the control

The objective of this study was to evaluate the efficacy of complete edible coating formulations enriched with essential oils as practical postharvest preservation systems rather than to investigate the individual contribution of each coating component. Similar formulation-based approaches have been widely adopted in studies evaluating edible coatings for fresh produce preservation, where the performance of the integrated coating system represents the primary focus^16,17^. Therefore, treatments containing the coating matrices alone, without essential oil incorporation, were not included in the experimental design.

The selected biopolymer–essential oil combinations were chosen based on previous studies demonstrating their suitability for edible coating applications and postharvest preservation. Whey protein has been widely reported as an effective film-forming matrix with excellent oxygen barrier and emulsifying properties, facilitating the incorporation and controlled release of essential oils such as lemon oil^18,19^. GA possesses superior emulsification capacity and coating stability, making it an appropriate carrier for bioactive compounds including peppermint oil^20,21^. Likewise, (CMC) was selected because of its excellent film-forming ability, transparency, flexibility, and moisture barrier properties, while clove oil was incorporated due to its strong antioxidant and antimicrobial activity^22,23^. Therefore, these formulations were selected as representative functional coating systems to evaluate their effectiveness in preserving the quality and storage stability of fresh-cut artichoke.

#### Methods for extracting essential oils

In the laboratory of the Department of postharvest and handling Vegetables Research, Horticultural Research Institute, Agricultural Research Center, Giza, Egypt, 20 g of mint leaves, clove flower buds, and lemon fruit peel were taken after cleaning and drying. The plant material was procured from a certified local herbal supplier. The essential oil was extracted by steam distillation using a Clevenger-type apparatus for 3–4 h. The obtained oil was then dried over anhydrous sodium sulfate, filtered, and stored in amber glass bottles at 4 °C until further analysis to prevent oxidation and volatilization of its active compounds.

There were three methods of storage used:

1. The refrigeration stage at 0 °C with 95% relative humidity,
2. Individual Quick Freezing (IQF) at -30 °C.
3. The two techniques are combined: IQF at -30 °C and thawing at 0 °C in the refrigeration stage with 95% relative humidity.

The qualities listed below were recorded after a two-week storage period for each treatment. Approximately 1000 kg of freshly harvested artichokes were used throughout the entire study. The material was subdivided among all coating, packaging, and storage treatments. Each treatment consisted of three independent biological replicates, with each replicate containing approximately 1 kg of fresh-cut artichoke packed separately in an individual package. The package was considered the experimental unit for statistical analysis.

### The total carbohydrates

The total carbohydrate content of the sample is determined using the colorimetric phenol-sulfuric acid method, as outlined by^24^.

### Protein content

Protein determined using the Kjeldahl method outlined by^25^.

### Total sugars

The concentration of the total sugars in a sample can be determined using the colorimetric approach known as the Anthrone method. The anthrone reagent interacts with sugars in an acidic environment to produce a blue-green hue. The anthrone reagent and sulfuric acid are combined with the sample, which is then cooked until the reaction is finished. Next, the mixture is allowed to cool, and at 620 nm, its absorbance is determined. The amount of sugar in the original sample and the absorbance are linearly correlated^26^.

### Folic acid

Spectrophotometric technique was created to measure folic acid by^27^.

### Inulin content

The inulin was extracted from the artichoke tuber samples using an accelerated solvent extraction technique. After that, spectrophotometry was used to determine the amount of fructose in the hydrolysates. The spectrophotometric technique is based on measuring the absorbance at 350 nm of the triiodide complex produced by the periodate oxidation of fructose, upon the addition of potassium iodide^28^.

### Cynarin content

Cynarin determined according to^29^.

### Total phenols

The total Phenolic concentration was calculated using methodologies developed by^30,31^.

$${\mathrm{Total}}\:{\mathrm{phenolics}}\:{\mathrm{in}}\:{\mathrm{mg}}\:{\mathrm{GAE}}\:{\mathrm{per}}\:{\mathrm{g}}\:{\mathrm{of}}\:{\mathrm{dry}}\:{\mathrm{sample}} = \frac{{C \times DF}}{{{\mathrm{W}} \times 1000}}$$

Where: C= Concentration obtained from the calibration in µg/ml, DF= Total dilution factor; W= weight of the sample in grams, 1000 = conversion from µg/ml to mg/ml.

### Ascorbic acid (vit. C)

The vitamin C content is determined using UV spectroscopy, which measures the amount of vitamin C in a sample. According to^32^.

### Polyphenol oxidase (PPO) enzyme activity

Polyphenol oxidase (PPO) activity was determined according to the method of^33^ with slight modifications. Fresh tissue (1 g) was homogenized in 10 mL of cold phosphate buffer (0.1 M, pH 6.8) and centrifuged at 10,000 ×g for 15 min at 4 °C. The reaction mixture contained 2.9 mL of catechol solution (0.1 M) prepared in phosphate buffer and 0.1 mL of enzyme extract. The increase in absorbance at 420 nm was recorded for 3 min using a UV–Vis spectrophotometer. One unit of PPO activity was defined as the change in absorbance of 0.001 per minute under the assay conditions and expressed as U g⁻¹ fresh weight.

### Malondialdehyde (lipid oxidation)

The MDA concentration was determined using an extinction coefficient of 155 mM^− 1^ cm^− 1^^34^ after deducting the absorbance at 600 nm from the absorbance at 532 nm.

### Superoxide anion (O_2_^−^)

The superoxide anion (O_2_^−^) Fifty milligrams of the leaf samples were incubated in an extraction medium made up of 100 µM ethylenediaminetetraacetic acid (EDTA) disodium salt, 20 µM nicotinamide adenine dinucleotide (NADH), and 20 mM sodium phosphate buffer at pH 7.8 to measure the superoxide anion (O_2_^−^) concentration^35^. Adding 100 µL of 25.2 mM epinephrine in 0.1 N HCl started the reaction. The absorbance was measured at 480 nm for 5 min after the samples had been incubated for 5 min at 28 °C with continuous mixing. The buildup of adrenochrome was determined using a molar absorption coefficient of 4.0 × 103 M^− 1^^36^ to calculate superoxide anion production.

### Hydrogen peroxide content

Using 300 mg of samples that were homogenized in an extraction medium composed of 50 mM potassium phosphate buffer at pH 6.5 and 1 mM hydroxylamine, the hydrogen peroxide content (H_2_O_2_) concentration was ascertained. The mixture was then centrifuged for 15 min at 10,000×g at 4 °C. After that, 30 µL samples of the supernatant were added to a reaction medium containing 100 µM FeNH_4_SO_4_, 25 mM sulfuric acid, 250 µM xylenol orange, and 100 mM sorbitol^37^. The absorbance of the samples was measured at 560 nm after they were kept in the dark for 30 min.

### Determination of Potassium

It was estimated Flame photometrically using Ienway Flame photometer model Corning 400 according to^38^.

### Determination of Fe

Using the method described by^39^, the heavy metal content was examined using the Perkin Elmer Model 5100 electrothermal atomic absorption spectrometer.

### Texture

The texture of the sample was measured using a TA.XT2i Plus texture analyzer (Stable Micro Systems Ltd., Godalming, Surrey, UK). Fresh-cut artichoke was placed on the test platform with the skin facing upward. The probe (P/2, 2 mm cylindrical probe) was compressed to 50% of the total sample height at a pre-test speed of 2 mm/s, a test speed of 1 mm/s, and a post-test speed of 2 mm/s. The maximum force was recorded as the hardness of the sample, while the number of peaks represented the sample fragility (brittleness).

### Weight loss percent

The formula for the percentage of weight reduction (WLP %) used by^40^ [(initial – final weight)/initial weight] × 100 was used to calculate the WLP% following the storage period.

### Ethylene

Ethylene gas determined according to^41^.

### Respiration rate

Respiratory rate a gas-tight container of known volume is filled with product in the closed system, and the container, which initially contained ambient air as its atmosphere, is closed. The changes in CO_2_ concentration over a specific period are measured and used to calculate respiration rates^42^.

### Microorganism

#### Pathogenic strains including

##### Bacteria

The bacterial strains used in this investigation (Gram positive and Gram negative) were kindly supplied by the Microbiology Department, Faculty of Agricultural Cairo University. These strains were *Staphylooccus aureus*, ATTCC 25,923, *E.* coli, ATCC 25,922, *Bacillus cereus* ATCC 33,018 Cultures were maintained on nutrient agar slants (4 °C) and sub cultured at 37 °C in nutrient broth for 24 h prior to incubation.

##### Fungi

Isolates of fungi were *Aspergillus niger* and obtained from Microbiology Center (CATM), Ain-Shams Uni.

##### Media used

All media consist of (gL^− 1^). Plate count agar medium, Potato dextrose agar medium and Nutrient Agar medium.

### Microbiological methods

Total count bacteria were determined on plate count agar according to^43^.

Yeast and mold were enumerated using Potato dextrose agar according to^44^.

### Determination of antibacterial and antifungal activity

For the antibacterial activity test, prepared coating solution was kept in a 100 mL beaker and sterile filter paper discs were immersed in the solution. Antibacterial and antifungal activities of the coating formulations were evaluated against the selected bacterial and fungal strains using the disc diffusion method according to^45^. The plates were incubated at 35 °C overnight for bacteria and at 27 °C for 3 days for fungi. Inhibition zones were measured and expressed in (mm). Antimicrobial activity was evaluated using the edible coating formulations themselves rather than the dried films or coated artichoke samples. The disc diffusion assay was performed to assess the inhibitory potential of each coating solution containing the respective biopolymer matrix and essential oil components against selected bacterial and fungal strains. Therefore, the recorded inhibition zones represent the antimicrobial efficacy of the coating formulations under in vitro conditions. In contrast, microbial counts determined during storage were used to evaluate the effectiveness of coated and packaged artichoke samples in suppressing microbial proliferation under practical storage conditions. Two commercially available polymeric film bags were used as packaging materials. The investigated polymer materials were low-density polyethylene (LDPE) 20 × 30 cm of 40 μm thickness and polypropylene (BOPP Biaxial oriented) 20 × 30 cm of 24 μm thickness. The average oxygen transmission rates (OTR)of the PE and PP films are 4000 cc ∕ m^2^ ∕ day and 1000 cc ∕ m^2^ ∕ day ‚while the water vapor transmission rates (WVPR) of films is LDPE 20 gm ∕ m^2^ ∕ day and 70 gm ∕ m^2^ ∕ day respectively.

### Characterization of edible films

#### Film thickness

Film thickness was measured using a Mitutoyo micrometer (Model MDC-25 M, MFG / Japan). The final value represented the average of 10 random measurements taken at different parts of the film as reported by (R)^46^.

#### Water vapor transmission

The water vapor transmission rate [g/ (s.m^2^)] and water vapor permeability (WVP) through films were determined gravimetrically using the^47^ Method E96-95. A circular test cup was used to determine the WVP of the film. The film was first cut into circular shape that was larger than the inner diameter of the cup, the cup was filled with 50% distilled water and the film was sealed at the top using paraffin oil, then the cups were placed in a desiccator containing anhydrous calcium chloride. The weights of the cups were recorded every hour during 10 h and to specimens of each film were tested. Linear regression was used to estimate the slope of this line in g/h. The water vapor transmission rate (WVPR) and water vapor permeability were determined using the following equation:

$${\mathrm{WVPR}} = \Delta {\mathrm{m}}/\Delta {\mathrm{tA}}$$

Where, **∆m**/**∆t** is the moisture gain weight per time (g/s), A is the surface area of the film (m^2^) L is the film thickness (mm) and is the difference in relative humidity^48^.

### Transparency of edible films

Transparency acts as an additional factor to exploit the constituents of edible film^49^. Transmittance to visible light was measured at wavelength 550 nm.

### Water solubility of edible films

The solubility of different edible films was determined according to method given by^50^. The film solubility expresses the percentage of the films dry matter solubilized after immersion of the per-weighted films (2 × 3 cm) in 80 ml of distilled water for 24 h at room temperature with constant agitation. The remained pieces of the film after immersion filtered using filter paper, were dried at 60 °C in an oven constant weight. Film solubility(S%) was calculated from the following equation:

$${\mathrm{Solubility}}\% = \frac{{{\mathrm{Initial}}\;{\mathrm{dry}}\;{\mathrm{weight}} - {\mathrm{final}}\;{\mathrm{dry}}\;{\mathrm{weight}} \times 100}}{{{\mathrm{Initial}}\;{\mathrm{dry}}\;{\mathrm{weight}}}}$$

### Statistical analysis

The experiment was conducted as a factorial arrangement consisting of four coating treatments (control, whey protein + lemon oil, GA + Peppermint oil, and CMC + clove oil), three storage conditions (cooling, IQF, and cooling + IQF), and two packaging materials (polyethylene and polypropylene). Each treatment combination was replicated three times. One package containing 1 kg of fresh-cut artichoke represented one biological replicate and was considered the experimental unit for statistical analysis.

The experiment was arranged as a completely factorial design consisting of three factors: (i) edible coating treatment with four levels [control, whey protein + lemon oil, GA + Peppermint oil, and CMC + clove oil], (ii) storage condition with three levels [cooling at 0 °C, (IQF) at -30 °C, and cooling + IQF], and (iii) packaging material with two levels [polyethylene (PE) and polypropylene (PP)]. Therefore, a total of 24 treatment combinations (4 × 3 × 2) were evaluated. Each treatment combination was replicated three times, and each replicate package was considered an independent experimental unit.

Data were analyzed using a three-factor factorial ANOVA considering coating treatment, storage condition, and packaging material as fixed factors “COSTATE” program version (6.400) as reported by ^51^. Main effects and all possible interactions were evaluated. Mean separation was performed using Duncan’s multiple range test at *P* ≤ 0.05. Results are presented as mean ± standard deviation of three independent biological replicates.

## Results

To facilitate interpretation of the results, Table 1 summarizes all tables included in the study along with their main measured parameters and experimental focus.

**Table 1:** All tables included in the study along with their main measured parameters and experimental focus.

Table No.	Parameters evaluated	Best performing treatment	Main finding
Table 2	Total carbohydrates, protein	CMC + clove oil + IQF	Highest retention of carbohydrates and protein
Table 3	Total sugars, folic acid	CMC + clove oil	Maximum preservation of sugars and folic acid
Table 4	Inulin, cynarin	CMC + clove oil + IQF	Highest retention of bioactive compounds
Table 5	Total phenols, vitamin C	GA + Peppermint oil/CMC + clove oil	Improved antioxidant preservation
Table 6	PPO activity, MDA	CMC + clove oil	Lowest oxidative damage indicators
Table 7	H₂O₂, superoxide radical	CMC + clove oil	Significant reduction in oxidative stress
Table 8	Potassium, iron	IQF treatments	Improved mineral retention
Table 9	Texture, weight loss	GA + Peppermint oil/CMC + clove oil	Improved firmness and reduced weight loss
Table 10	Ethylene, respiration rate	CMC + clove oil	Reduced metabolic activity
Table 11	Microbial counts (PP)	CMC + clove oil	Lowest bacterial and fungal counts
Table 12	Microbial counts (PE)	Coated treatments	Reduced microbial growth vs. control
Table 13	Antimicrobial activity	Whey protein + lemon oil	Largest inhibition zones
Table 14	Film properties	All treatments	Comparative evaluation of mechanical and barrier properties

**Table 2:** Mean and standard deviation of Edible coatings, storage conditions and their interaction on total carbohydrate percent and protein percent of artichoke fresh cut with polyethylene and polypropylene packages after storage for two weeks during both seasons 2024 and 2025.

Edibles coating B	Storage conditions A							
	Polyethylene package							
	Total carbohydrate percent				Protein percent			
	Cooling (0 °C)	IQF (-30 °C)	Chilling (0 °C) + IQF (-30 °C)	Mean B	Cooling (0 °C)	IQF (-30 °C)	Chilling (0 °C) + IQF (-30 °C)	Mean B
Control	17.55±0.23	20.35±0.26	18.12±0.25	17.95c	13.69±0.20	13.92±0.06	13.52±0.17	15.40a
Whey protein+ Lemon oil	18.45±0.26	21.18±0.31	19.53±0.27	20.11a	16.92±0.21	17.30±0.25	16.48±0.23	15.24a
GA + Peppermint oil	17.83±0.23	20.74±0.29	19.18±0.25	20.18a	15.60±0.21	16.05±0.20	15.20±0.19	14.80b
CMC+ Clove oil	18.81±0.26	21.67±0.28	19.92±0.28	19.55b	14.50±0.19	14.82±0.20	14.18±0.07	15.29a
Mean A	19.16b	19.91a	19.26b		15.18a	15.21a	15.15a	
LSD 0.05	A=0.27 B=0.23 A×B=0.47				A=0.19 B=0.15 A×B=0.33			
F- test	A= ** B= ** A×B=**				A= ** B= ** A×B=**			
P value	A= 0.00001 B= 0.00001 A×B=0.00001				A= 0.00001 B= 0.00001 A×B=0.00001			

Edibles coating B	Storage Conditions A							
	Polypropylene package							
	Total carbohydrate percent				Protein percent			
	Cooling (0 °C)	IQF (-30 °C)	Chilling (0 °C) + IQF (-30 °C)	Mean B	Cooling (0 °C)	IQF (-30 °C)	Chilling (0 °C) + IQF (-30 °C)	Mean B
Control	15.98±0.23	18.71±0.09	16.63±0.21	16.39d	12.43±0.16	12.66±0.18	12.25±0.15	14.13a
Whey protein+ Lemon oil	17.15±0.22	19.10±0.28	17.87±0.25	18.49b	15.59±0.21	15.94±0.20	15.09±0.21	13.97b
GA + Peppermint oil	16.04±0.22	19.64±0.25	17.82±0.23	18.83a	14.37±0.18	14.65±0.21	13.98±0.18	13.47c
CMC+ Clove oil	17.65±0.23	20.22±0.28	18.26±0.09	17.99c	13.32±0.19	13.50±0.19	12.86±0.06	14.13b
Mean A	17.79b	18.48a	17.51c		13.88a	13.93a	13.85a	
LSD 0.05	A=0.18 B=0.24 A×B=0.42				A=0.11 B=0.15 A×B=0.20			
F- test	A= ** B= ** A×B=**				A= ** B= ** A×B=**			
P value	A= 0.00001 B= 0.00001 A×B=0.00001				A= 0.00001 B= 0.00001 A×B=0.00001			

*p*>0.05 (non-significant), *p*≤0.05 (significant), *p*≤0.01 (high significant), *p*≤0.001 (very high significant), *p*≤0.0001(very very high significant), GA, gum Arabic, CMC, carboxymethyl cellulose, IQF, Individual Quick Freezing.

**Table 3:** Mean and standard deviation of Edible coatings, storage conditions and their interaction on total sugar percent and folic acid (µg/100 g)of artichoke fresh cut with polyethylene and polypropylene packages after storage for two weeks during both seasons 2024 and 2025.

Edibles Coating B	Storage Conditions A									
	Polyethylene package									
	Total sugar percent				Folic acid ( µg/100 g)					
	Cooling (0 °C)	IQF (-30 °C)	Chilling (0 °C) + IQF (-30 °C)	Mean B	Cooling (0 °C)	IQF (-30 °C)		Chilling (0 °C) IQF (-30 °C)		Mean B
Control	6.32±0.10	7.48±0.11	6.55±0.09	6.37c	23.50±0.80	38.40±0.90		29.17±0.75		30.36b
Whey protein+ Lemon oil	6.69±0.09	7.88±0.10	7.13±0.10	7.39a	17.27±0.65	35.77±1.25		25.27±0.65		26.10c
GA + Peppermint oil	6.10±0.51	7.67±0.11	6.98±0.09	7.43a	18.67±0.65	33.47±1.15		27.17±0.65		30.68a
CMC+ Clove oil	6.82±0.09	8.08±0.11	7.30±0.10	7.14b	21.77±0.75	31.30±1.00		20.17±0.55		24.41d
Mean A	6.99b	7.31a	6.96b		24.44c	30.09a		25.94b		
LSD 0.05	A=0.25 B=0.14 A×B=0.43				A=0.32 B=0.79 A×B=0.56					
F- test	A= ** B= ** A×B=**				A= ** B= ** A×B=**					
P value	A= 0.00001 B= 0.00001 A×B=0.00001				A= 0.00001 B= 0.00001 A×B=0.00001					

Edibles Coating B	Storage Conditions A									
	Polypropylene package									
	Total sugar percent				Folic acid ( µg/100 g)					
	Cooling (0 °C)	IQF (-30 °C)	Chilling (0 °C) + IQF (-30 °C)	Mean B	Cooling (0 °C)	IQF (-30 °C)	Chilling (0 °C) + IQF (-30 °C)		Mean B	
Control	5.57±0.07	6.74±0.09	5.86±0.08	5.77d	20.88±0.74	33.27±0.81	25.36±0.65		26.50b	
Whey protein+ Lemon oil	5.99±0.08	7.01±0.10	6.33±0.08	6.59b	14.99±0.51	29.25±0.67	22.52±0.57		22.25c	
GA + Peppermint oil	5.75±0.07	6.95±0.09	6.27±0.08	6.68a	16.29±0.54	31.01±0.80	23.79±0.55		27.36a	
CMC+ Clove oil	6.01±0.09	7.23±0.09	6.66±0.09	6.42c	16.54±0.66	26.59±0.90	17.79±0.43		20.31d	
Mean A	6.21c	6.56a	6.32b		21.99b	26.13a	22.17b			
LSD 0.05	A=0.09 B=0.07 A×B=0.15				A=0.80 B=0.56 A×B=1.39					
F- test	A= ** B= ** A×B=**				A= ** B= ** A×B=**					
P value	A= 0.00001 B= 0.00001 A×B=0.00001				A= 0.00001 B= 0.00001 A×B=0.00001					

*p*>0.05 (non-significant), *p*≤0.05 (significant), *p*≤0.01 (high significant), *p*≤0.001 (very high significant), *p*≤0.0001(very very high significant), GA, gum Arabic, CMC, carboxymethyl cellulose, IQF, Individual Quick Freezing.

**Table 4:** Mean and standard deviation of Edible coatings, storage conditions and their interaction on inulin percent and cynarin percent of artichoke fresh cut with polyethylene and polypropylene packages after storage for two weeks during both seasons 2024 and 2025.

Edibles Coating B	Storage Conditions A									
	Polyethylene package									
	Inulin percent				Cynarin percent					
	Cooling (0 °C)	IQF (-30 °C)	Chilling(0 °C) + IQF (-30 °C)	Mean B	Cooling (0 °C)	IQF (-30 °C)		Chilling (0 °C)+ IQF (-30 °C)		Mean B
Control	7.64±0.11	9.09±0.14	7.77±0.11	7.89c	0.75±0.04	1.25±0.04		0.85±0.03		0.82d
Whey protein+ Lemon oil	7.92±0.11	9.31±0.12	8.46±0.12	8.89a	0.80±0.07	1.32±0.04		1.02±0.04		1.18b
GA + Peppermint oil	8.10±0.11	9.48±0.15	8.67±0.11	8.99a	0.90±0.08	1.40±0.04		1.09±0.06		1.25a
CMC+ Clove oil	8.27±0.11	9.71±0.14	8.87±0.12	8.67b	0.96±0.05	1.51±0.08		1.16±0.04		1.09c
Mean A	8.46b	8.85a	8.52b		1.03b	1.16a		1.06b		
LSD 0.05	A=0.15 B=0.08 A×B=0.27				A=0.07 B=0.04 A×B=0.11					
F- test	A= ** B= ** A×B=**				A= ** B= ** A×B=**					
P value	A= 0.00001 B= 0.00001 A×B=0.00001				A= 0.00001 B= 0.00001 A×B=0.00001					

Edibles Coating B	Storage Conditions A									
	Polypropylene package									
	Inulin percent				Cynarin percent					
	Cooling (0 °C)	IQF (-30 °C)	Chilling(0 °C) + IQF (-30 °C)	Mean B	Cooling (0 °C)	IQF (-30 °C)	Chilling (0 °C)+ IQF (-30 °C)		Mean B	
Control	6.98±0.11	7.99±0.12	7.01±0.10	7.11d	0.65±0.04	1.07±0.04	0.75±0.02		0.71d	
Whey protein+ Lemon oil	6.98±0.09	8.47±0.11	7.60±0.10	7.98b	0.70±0.06	1.15±0.04	0.88±0.05		1.03b	
GA + Peppermint oil	7.37±0.09	8.58±0.13	7.65±0.09	8.12a	0.79±0.07	1.23±0.04	0.96±0.03		1.09a	
CMC+ Clove oil	7.48±0.11	8.77±0.13	8.15±0.11	7.80c	0.86±0.03	1.30±0.04	1.01±0.03		0.95c	
Mean A	7.66c	7.85a	7.75b		0.91b	1.01a	0.93b			
LSD 0.05	A=0.14 B=0.80 A×B=0.23				A=0.50 B=0.30 A×B=0.80					
F- test	A= ** B= ** A×B=**				A= ** B= ** A×B=**					
P value	A= 0.00001 B= 0.00001 A×B=0.00001				A= 0.00001 B= 0.00001 A×B=0.00001					

*p*>0.05 (non-significant), *p*≤0.05 (significant), *p*≤0.01 (high significant), *p*≤0.001 (very high significant), *p*≤0.0001(very very high significant), GA, gum Arabic, CMC, carboxymethyl cellulose, IQF, Individual Quick Freezing.

**Table 5:** Mean and standard deviation of Edible coatings, storage conditions and their interaction on total phenols (mg GAE.g^-1^ and Vit. C (mg.100 g^-1^ of artichoke fresh cut with polyethylene and polypropylene packages after storage for two weeks in during both seasons 2024 and 2025.

Edibles Coating B	Storage Conditions A									
	Polyethylene package									
	Total phenols (mg GAE.g^-1^)				Vit. C (mg.100 g^-1^)					
	Cooling (0 °C)	IQF (-30 °C)	Chilling (0 °C)+ IQF (-30 °C)	Mean B	Cooling (0 °C)	IQF (-30 °C)		Chilling(0 °C) + IQF (-30 °C)		Mean B
Control	22.17±0.35	33.35±0.48	23.38±0.33	23.68d	11.63±0.16	11.07±0.16		10.60±0.14		11.10a
Whey protein+ Lemon oil	23.22±0.35	35.22±0.50	28.45±0.35	31.84b	8.36±0.11	9.81±0.13		8.92±0.12		9.03c
GA + Peppermint oil	25.65±0.35	37.17±0.50	31.65±0.40	33.62a	9.49±0.12	9.18±0.14		8.25±0.10		8.97d
CMC+ Clove oil	26.97±0.40	39.32±0.48	29.97±0.40	30.02c	8.70±0.13	8.51±0.12		10.17±0.46		9.13b
Mean A	28.69b	31.88a	28.80b		8.79c	10.45a		9.43b		
LSD 0.05	A=0.55 B=0.18 A×B=0.94				A=0.08 B=0.07 A×B=0.14					
F- test	A= ** B= ** A×B=**				A= ** B= ** A×B=**					
P value	A= 0.00001 B= 0.00001 A×B=0.00001				A= 0.00001 B= 0.00001 A×B=0.00001					

Edibles Coating B	Storage Conditions A									
	Polypropylene package									
	Total phenols (mg GAE.g^-1^)				Vit. C (mg.100 g^-1^)					
	Cooling (0 °C)	IQF (-30 °C)	Chilling (0 °C) + IQF (-30 °C)	Mean B	Cooling (0 °C)	IQF (-30 °C)	Chilling (0 °C) + IQF (-30 °C)		Mean B	
Control	19.35±0.30	27.41±0.41	20.67±0.27	20.34d	7.81±0.12	9.05±0.13	9.50±0.12		8.79a	
Whey protein+ Lemon oil	20.25±0.26	29.53±0.42	23.83±0.34	26.63b	6.30±0.13	8.24±0.11	7.34±0.10		7.73b	
GA + Peppermint oil	21.42±0.33	30.68±0.46	26.58±0.35	27.76a	7.61±0.09	8.07±0.12	7.37±0.09		7.68c	
CMC+ Clove oil	22.95±0.30	31.94±0.46	24.60±0.32	25.00c	6.94±0.09	7.14±0.10	8.40±0.04		7.49d	
Mean A	24.20b	26.55a	24.05b		7.54c	8.75a	7.91b			
LSD 0.05	A=0.49 B=0.15 A×B=0.86				A=0.07 B=0.08 A×B=0.17					
F- test	A= ** B= ** A×B=**				A= ** B= ** A×B=**					
P value	A= 0.00001 B= 0.00001 A×B=0.00001				A= 0.00001 B= 0.00001 A×B=0.00001					

*p*>0.05 (non-significant), *p*≤0.05 (significant), *p*≤0.01 (high significant), *p*≤0.001 (very high significant), *p*≤0.0001(very very high significant), GA, gum Arabic, CMC, carboxymethyl cellulose, IQF, Individual Quick Freezing.

**Table 6:** Mean and standard deviation of Edible coatings, storage conditions and their interaction on poly phenol oxidase (ΔA.min^-1^.g^-1^ and malondialdehyde (µmol.g^-1^ F.W) of artichoke fresh cut with polyethylene and polypropylene packages after storage for two weeks during both seasons 2024 and 2025.

Edibles Coating B	Storage Conditions A									
	Polyethylene package									
	Poly phenol oxidase (ΔA.min^-1^.g^-1^)				Malondialdehyde (µmol.g^-1^ F.W)					
	Cooling (0 °C)	IQF (-30 °C)	Chilling (0 °C) + IQF (-30 °C)	Mean B	Cooling (0 °C)	IQF (-30 °C)		Chilling (0 °C) + IQF (-30 °C)		Mean B
Control	0.59±0.03	0.27±0.01	0.50±0.01	0.53a	18.01±0.25	13.30±0.19		16.69±0.24		17.13a
Whey protein+ Lemon oil	0.54±0.04	0.24±0.01	0.38±0.02	0.31bc	17.34±0.24	12.80±0.20		14.89±0.20		13.86c
GA + Peppermint oil	0.46±0.04	0.21±0.01	0.34±0.01	0.30c	16.06±0.24	12.33±0.19		14.33±0.22		13.64d
CMC+ Clove oil	0.42±0.01	0.18±0.01	0.30±0.01	0.34b	15.47±0.23	11.89±0.17		13.81±0.19		14.34b
Mean A	0.40a	0.33c	0.37b		15.17a	14.21c		14.84b		
LSD 0.05	A=0.30 B=0.10 A×B=0.06				A=0.21 B=0.19 A×B=0.36					
F- test	A= ** B= ** A×B=**				A= ** B= ** A×B=**					
P value	A= 0.00001 B= 0.00001 A×B=0.00001				A= 0.00001 B= 0.00001 A×B=0.00001					

Edibles Coating B	Storage Conditions A									
	Polypropylene package									
	Poly phenol oxidase (ΔA.min^-1^.g^-1^)				Malondialdehyde (µmol.g^-1^ F.W)					
	Cooling (0 °C)	IQF (-30 °C)	Chilling (0 °C) + IQF (-30 °C)	Mean B	Cooling (0 °C)	IQF (-30 °C)	Chilling (0 °C) + IQF (-30 °C)		Mean B	
Control	0.67±0.06	0.31±0.01	0.55±0.03	0.61a	21.85±0.28	15.88±0.23	20.12±0.26		20.60a	
Whey protein+ Lemon oil	0.62±0.05	0.27±0.01	0.42±0.02	0.35bc	21.27±0.31	14.84±0.20	18.45±0.09		16.40c	
GA + Peppermint oil	0.54±0.02	0.24±0.01	0.39±0.01	0.33c	18.69±0.26	14.61±0.18	17.56±0.23		16.19d	
CMC+ Clove oil	0.48±0.02	0.20±0.00	0.35±0.01	0.39b	18.49±0.24	13.83±0.19	17.14±0.23		17.72b	
Mean A	0.45a	0.38c	0.43b		18.35a	17.13c	17.70b			
LSD 0.05	A=0.40 B=0.22 A×B=0.31				A=0.20 B=0.21 A×B=0.35					
F- test	A= ** B= ** A×B=**				A= ** B= ** A×B=**					
P value	A= 0.00001 B= 0.00001 A×B=0.00001				A= 0.00001 B= 0.00001 A×B=0.00001					

*p*>0.05 (non-significant), *p*≤0.05 (significant), *p*≤0.01 (high significant), *p*≤0.001 (very high significant), *p*≤0.0001(very very high significant), GA, gum Arabic, CMC, carboxymethyl cellulose, IQF, Individual Quick Freezing.

**Table 7:** Mean and standard deviation of Edible coatings, storage conditions and their interaction on hydrogen peroxide content (µmol.g^-1^ F.W) and superoxide content (µmol.g^-1^ F.W) of artichoke fresh cut with polyethylene and polypropylene packages after storage for two weeks during both seasons 2024 and 2025.

Edibles coating B	Storage Conditions A									
	Polyethylene package									
	Hydrogen peroxide content (µmol.g^-1^ F.W)				Superoxide content (µmol.g^-1^ F.W)					
	Cooling (0 °C)	IQF (-30 °C)	Chilling (0 °C) + IQF (-30 °C)	Mean B	Cooling (0 °C)	IQF (-30 °C)		Chilling (0 °C) + IQF (-30 °C)		Mean B
Control	1.26±0.02	0.89±0.01	1.15±0.02	1.19a	1.44±0.02	0.98±0.01		1.30±0.02		1.35a
Whey protein+ Lemon oil	1.21±0.02	0.85±0.01	1.01±0.01	0.93c	1.37±0.02	0.94±0.01		1.13±0.02		1.03c
GA + Peppermint oil	1.09±0.02	0.82±0.01	0.97±0.01	0.92c	1.24±0.02	0.90±0.01		1.08±0.01		1.02d
CMC+ Clove oil	1.05±0.02	0.79±0.01	0.93±0.01	0.97b	1.18±0.02	0.86±0.01		1.03±0.01		1.08b
Mean A	1.04a	0.96c	1.00b		1.16a	1.07c		1.13b		
LSD 0.05	A=0.10 B=0.21 A×B=0.13				A=0.11 B=0.81 A×B=0.26					
F- test	A= ** B= ** A×B=**				A= ** B= ** A×B=**					
P value	A= 0.00001 B= 0.00001 A×B=0.00001				A= 0.00001 B= 0.00001 A×B=0.00001					

Edibles coating B	Storage Conditions A									
	Polypropylene package									
	Hydrogen peroxide content (µmol.g^-1^ F.W)				Superoxide content (µmol.g^-1^ F.W)					
	Cooling (0 °C)	IQF (-30 °C)	Chilling (0 °C) + IQF (-30 °C)	Mean B	Cooling (0 °C)	IQF (-30 °C)	Chilling (0 °C) + IQF (-30 °C)		Mean B	
Control	1.54±0.02	1.02±0.01	1.35±0.02	1.45a	1.66±0.02	1.08±0.01	1.51±0.02		1.57a	
Whey protein+ Lemon oil	1.50±0.02	1.01±0.01	1.21±0.02	1.10c	1.60±0.02	1.07±0.01	1.30±0.02		1.18c	
GA + Peppermint oil	1.32±0.02	0.96±0.01	1.21±0.02	1.06d	1.45±0.02	1.00±0.01	1.20±0.02		1.16d	
CMC+ Clove oil	1.27±0.02	0.88±0.01	1.09±0.02	1.17b	1.40±0.02	0.95±0.01	1.16±0.02		1.22b	
Mean A	1.24a	1.15c	1.19b		1.34a	1.20c	1.30b			
LSD 0.05	A=0.25 B0.21 A×B=0.13				A=0.22 B=0.32 A×B=0.83					
F- test	A= ** B= ** A×B=**				A= ** B= ** A×B=**					
P value	A= 0.00001 B= 0.00001 A×B=0.00001				A= 0.00001 B= 0.00001 A×B=0.00001					

*p*>0.05 (non-significant), *p*≤0.05 (significant), *p*≤0.01 (high significant), *p*≤0.001 (very high significant), *p*≤0.0001(very very high significant), gum Arabic, CMC, carboxymethyl cellulose, IQF, Individual Quick Freezing.

**Table 8:** Mean and standard deviation of Edible coatings, storage conditions and their interaction on potassium percent and iron (mg kg^-1^ of artichoke fresh cut with polyethylene and polypropylene packages after storage for two weeks during both seasons 2024 and 2025.

Edibles Coating B	Storage Conditions A									
	Polyethylene package									
	Potassium percent				Iron (mg.kg^-1^)					
	Cooling (0 °C)	IQF (-30 °C)	Chilling (0 °C) + IQF (-30 °C)	Mean B	Cooling (0 °C)	IQF (-30 °C)		Chilling (0 °C) + IQF (-30 °C)		Mean B
Control	1.78±0.05	2.08±0.05	1.84±0.04	1.82c	24.68±0.38	27.37±0.41		25.82±0.33		25.07c
Whey protein+ Lemon oil	1.81±0.05	2.13±0.06	1.95±0.04	2.04ab	24.99±0.32	27.81±0.40		26.24±0.38		27.03ab
GA + Peppermint oil	1.87±0.05	2.18±0.05	1.99±0.06	2.08a	25.55±0.40	28.23±0.42		26.64±0.35		27.41a
CMC+ Clove oil	1.91±0.05	2.23±0.06	2.03±0.05	1.99b	25.92±0.34	28.71±0.41		27.02±0.35		26.63b
Mean A	1.95b	2.03b	1.97b		26.27b	26.93a		26.41b		
LSD 0.05	A=0.11 B=0.40 A×B=0.09				A=0.53 B=0.18 A×B=0.91					
F- test	A= ** B= ** A×B=**				A= ** B= ** A×B=**					
P value	A= 0.00001 B= 0.00001 A×B=0.00001				A= 0.00001 B= 0.00001 A×B=0.00001					

Edibles Coating B	Storage Conditions A									
	Polypropylene package									
	Potassium percent				Iron (mg.kg^-1^)					
	Cooling (0 °C)	IQF (-30 °C)	Chilling (0 °C) + IQF (-30 °C)	Mean B	Cooling (0 °C)	IQF (-30 °C)	Chilling (0 °C) + IQF (-30 °C)		Mean B	
Control	1.66±0.06	1.94±0.06	1.76±0.05	1.72d	22.97±0.35	25.57±0.39	23.70±0.31		23.46c	
Whey protein+ Lemon oil	1.72±0.06	2.02±0.07	1.83±0.05	1.93b	23.36±0.30	26.51±0.38	24.68±0.36		25.53ab	
GA + Peppermint oil	1.78±0.06	2.09±0.07	1.87±0.05	1.99a	24.04±0.37	26.91±0.40	25.04±0.23		25.87a	
CMC+ Clove oil	1.84±0.07	2.12±0.05	1.91±0.05	1.87c	24.50±0.32	27.00±0.39	25.43±0.33		25.05b	
Mean A	1.85a	1.91a	1.87a		24.77b	25.24a	24.92b			
LSD 0.05	A=0.12 B=0.60 A×B=0.25				A=0.49 B=0.17 A×B=0.86					
F- test	A= ** B= ** A×B=**				A= ** B= ** A×B=**					
P value	A= 0.00001 B= 0.00001 A×B=0.00001				A= 0.00001 B= 0.00001 A×B=0.00001					

*p*>0.05 (non-significant), *p*≤0.05 (significant), *p*≤0.01 (high significant), *p*≤0.001 (very high significant), *p*≤0.0001(very very high significant), gum Arabic, CMC, carboxymethyl cellulose, IQF, Individual Quick Freezing.

**Table 9:** Mean and standard deviation of Edible coatings, storage conditions and their interaction on texture and weight loss percent of artichoke fresh cut with polyethylene and polypropylene packages after storage for two weeks during both seasons 2024 and 2025.

Edibles Coating B	Storage Conditions A									
	Polyethylene package									
	Texture				Weight loss percent					
	Cooling (0 °C)	IQF (-30 °C)	Chilling(0 °C) + IQF (-30 °C)	Mean B	Cooling (0 °C)	IQF (-30 °C)		Chilling (0 °C)+ IQF (-30 °C)		Mean B
Control	6.50±0.50	8.00±0.50	7.00±0.00	6.67d	6.62±0.46	10.50±1.47		12.69±1.20		9.94a
Whey protein+ Lemon oil	6.50±0.00	8.50±0.50	7.50±0.50	8.00b	2.28±0.53	2.33±0.61		2.28±1.04		5.44a
GA + Peppermint oil	7.00±0.50	9.00±0.50	8.00±0.50	8.33a	5.22±1.97	5.32±0.97		5.94±0.67		5.58a
CMC+ Clove oil	7.50±0.50	9.00±0.00	7.50±0.00	7.67c	3.49±0.78	6.72±1.18		8.49±1.22		5.57a
Mean A	7.63a	7.88a	7.50a		4.43b	6.36a		5.88a		
LSD 0.05	A=0.27 B=0.29 A×B=0.47				A=0.96 B=1.15 A×B=1.99					
F- test	A= ** B= ** A×B=**				A= ** B= ** A×B=**					
P value	A= 0.00001 B= 0.00001 A×B=0.00001				A= 0.00001 B= 0.00001 A×B=0.00001					

Edibles Coating B	Storage Conditions A									
	Polypropylene package									
	Texture				Weight loss percent					
	Cooling (0 °C)	IQF (-30 °C)	Chilling (0 °C) + IQF (-30 °C)	Mean B	Cooling (0 °C)	IQF (-30 °C)	Chilling (0 °C) + IQF (-30 °C)		Mean B	
Control	6.00±0.00	8.00±0.50	6.50±0.50	6.56d	8.46±1.84	7.67±2.07	7.88±1.63		6.46a	
Whey protein+ Lemon oil	6.67±0.29	8.00±0.50	7.00±0.00	7.67b	4.47±0.41	2.88±1.67	4.09±0.63		4.28b	
GA + Peppermint oil	7.00±0.50	9.00±0.00	7.00±0.50	8.17a	6.46±1.20	3.47±1.48	5.26±0.25		4.68b	
CMC+ Clove oil	7.00±0.50	9.00±0.50	7.00±0.50	7.00c	2.29±0.06	2.68±0.84	0.88±0.11		3.41b	
Mean A	7.25b	7.67a	7.13b		4.58a	5.02a	4.53a			
LSD 0.05	A=0.35 B=0.34 A×B=0.60				A=1.34 B=1.06 A×B=2.32					
F- test	A= ** B= ** A×B=**				A= ** B= ** A×B=**					
P value	A= 0.00001 B= 0.00001 A×B=0.00001				A= 0.00001 B= 0.00001 A×B=0.00001					

**Table 10:** Mean and standard deviation of Edible coatings, storage conditions and their interaction on ethylene (µl kg^-1^ h^-1^ and respiration rate (ml CO_2_ kg^-1^ h^-1^ of artichoke fresh cut with polyethylene an d polypropylene packages after storage for two weeks during both seasons 2024 and 2025.

Edibles coating B	Storage Conditions A									
	Polyethylene package									
	Ethylene (µl kg^-1^ h^-1^)				Respiration rate (ml CO_2_ kg^-1^ h^-1^)					
	Cooling (0 °C)	IQF (-30 °C)	Chilling(0 °C) + IQF (-30 °C)	Mean B	Cooling (0 °C)	IQF (-30 °C)		Chilling (0 °C) + IQF (-30 °C)	Mean B	
Control	3.11±0.05	1.55±0.03	2.66±0.03	2.81a	15.21±0.52	11.41±0.39		14.10±0.35	14.48a	
Whey protein+ Lemon oil	2.88±0.04	1.40±0.02	2.06±0.03	1.73c	14.62±0.52	11.04±0.25		12.66±0.33	11.86bc	
GA + Peppermint oil	2.45±0.04	1.26±0.02	1.88±0.02	1.68d	13.62±0.45	10.66±0.28		12.24±0.28	11.70c	
CMC+ Clove oil	2.25±0.03	1.13±0.02	1.71±0.02	1.88b	13.13±0.45	10.33±0.25		11.80±0.30	12.23b	
Mean A	2.17a	1.86c	2.05b		12.92a	12.15b		12.64a		
LSD 0.05	A=0.14 B=0. 20 A×B=0.25				A=0.33 B=0.39 A×B=0.67					
F- test	A= ** B= ** A×B=**				A= ** B= ** A×B=**					
P value	A= 0.00001 B= 0.00001 A×B=0.00001				A= 0.00001 B= 0.00001 A×B=0.00001					

Edibles coating B	Storage Conditions A									
	Polypropylene package									
	Ethylene (µl kg^-1^ h^-1^)				Respiration rate (ml CO_2_ kg^-1^ h^-1^)					
	Cooling (0 °C)	IQF (-30 °C)	Chilling (0 °C) + IQF (-30 °C)	Mean B	Cooling (0 °C)	IQF (-30 °C)	Chilling (0 °C) + IQF (-30 °C)			Mean B
Control	1.39±0.05	2.95±0.01	1.65±0.04	1.58d	12.51±0.16	16.65±0.24	13.52±0.17			13.11c
Whey protein+ Lemon oil	1.53±0.06	3.35±0.08	2.29±0.06	2.84b	12.53±0.18	17.01±0.24	15.21±0.07			15.84b
GA + Peppermint oil	1.82±0.06	3.47±0.09	2.50±0.06	2.95a	14.31±0.20	17.72±0.22	16.00±0.21			16.64a
CMC+ Clove oil	2.22±0.08	3.72±0.09	2.85±0.08	2.54c	13.85±0.18	16.68±0.26	16.80±0.25			16.00b
Mean A	2.34c	2.68a	2.42b		14.82c	15.97a	15.41b			
LSD 0.05	A=0.09 B=0.06 A×B=0.16				A=0.20 B=0.17 A×B=0.36					
F- test	A= ** B= ** A×B=**				A= ** B= ** A×B=**					
P value	A= 0.00001 B= 0.00001 A×B=0.00001				A= 0.00001 B= 0.00001 A×B=0.00001					

*p*>0.05 (non-significant), *p*≤0.05 (significant), *p*≤0.01 (high significant), *p*≤0.001 (very high significant), *p*≤0.0001(very very high significant), gum Arabic, CMC, carboxymethyl cellulose, IQF, Individual Quick Freezing.

**Table 11:** Effect of temperature on Total bacteria count (CFU x 10^-1^/ g) and Mold &yeast(10^1^CFU/g) of coated and uncoated artichokes in polypropylene) packaging)

Product property	Control	T1	T2	T3
Treatment at 0 °C (cooling)				
Total bacteria Count (10^1^CFU/g)	2.40±10^abcd^	2.30±10^abcd^	2.20±10^abcd^	2.39±10^abcd^
Mold &yeast (10^1^CFU/g)	2.30±10^abc^	2.01±10^abcd^	1.83±10^bcd^	2.17±10^abcd^
Treatment at -30 °C (IQF)				
Total bacteria Count (10^1^CFU/g)	1.40±10^abcd^	1.20±10^abcd^	ND	ND
Mold &yeast (10^1^CFU/g)	0.77±0.0.10^d^	0.83±10^d^	ND	ND
Treatment at 0°C + 30°C (cooling+ IQF)				
Total bacteria Count (10^1^CFU/g)	2.30±10^abcd^	1.13±10^abcd^	0.60±0.10^abcd^	0.76±0.10^d^
Mold &yeast (10^1^CFU/g)	1.30±10^abcd^	0.77±0.10^d^	0.86±0.10^d^	0.52±0.10^d^

There is a significant difference between treatment (means ±SD) for different capital letters in the same row and different lowercase letters in the same Colum at *p0.05.* T1: Whey protein+ Lemon oil, T2: GA, peppermint oil, T3: CMC+ Clove oil.

**Table 12:** Influence of storage temperature on microbial load of coated and uncoated artichokes in polyethylene packages.

Product property	Control	T1	T2	T3
Treatment at 0°C (cooling)				
Total bacteria Count (10^1^CFU/g)	2.03±10^abcd^	2.34±10^abcd^	2.14±10^abcd^	1.98±0.10^bcde^
Mold &yeast (10^1^CFU/g)	1.86±0.10^bc^	1.07±0.60^c^	0.66±10^c^	2.32±10^abc^
Treatment at -30°C (IQF)				
Total bacteria Count (10^1^CFU/g)	1.39±10c^de^	1.35±10^cde^	1.38±10^cde^	1.268±0.10^de^
Mold &yeast (10^1^CFU/g)	1.91±0.10^bc^	ND	1.40±0.10^bc^	1.30±0.10^bc^
Treatment at 0°C + 30°C (cooling+ IQF)				
Total bacteria Count (10^1^CFU/g)	0.80±0.10^de^	0.34±0.30^cde^	0.88±0.10de	0.56±0.10^e^
Mold &yeast (10^1^CFU/g)	0.66±0.10^c^	ND	1.67±10^bc^	ND

There is a significant difference between treatment (means ±SD) for different capital letters in the same row and different lowercase letters in the same Colum at *p0.05.* T1: Whey protein+ Lemon, T2: GA, peppermint oil, T3: Carboxymethyl cellulose+ Clove oil.

**Table 13:** Antimicrobial activity of (whey protein +Lemon oil &Arabic gum +peppermint oil &CMC+ clove oil) edible films with different ratio against pathogenic microorganisms.

Treatments	Organism	Inhibition diameter (mm)RM incorporation film
GA +peppermint oil whey protein +Lemon oil	*E. coli*	20 mm 24 mm
GA +peppermint oil whey protein +Lemon oil	*S.aureus*	20 mm 21 mm
GA +peppermint oil	*Bacills cereus*	8 mm
whey protein +Lemon oil	*Aspergillus niger*	8 mm

**Table 14:** Mechanical Properties, Permeability and Thickness of Prepared Produced edible coating and film.

Treatments	Thickness (mm)	Tensile Strength (MPa)	Elongation (%)	Water Vapor Transmission rate (g/h m²)	Solubility in water (%)	Trans Patency
1	0.57	0.84	36.30	69.767	87.44	44.13
2	0.12	1.56	3.55	71.04	79.68	2.70
3	0.36	0.16	26.9	43.61	47.76	5.23

T1: Whey protein+ Lemon, T2: GA+ peppermint oil, T3: CMC+ Clove oil.

### Effects of edible coatings, storage conditions, and packaging materials on nutritional quality

Analysis of variance revealed that edible coating treatments, storage conditions, and their interactions significantly affected all nutritional attributes of fresh-cut artichoke after two weeks of storage (*P* < 0.0001). Overall, samples packed in polyethylene exhibited superior preservation of nutritional compounds compared with polypropylene-packaged samples.

The application of edible coatings significantly enhanced total carbohydrates, proteins, total sugars, inulin, and mineral contents relative to the uncoated control as Tables 2, 3, 4 and 8. Among the tested coatings, whey protein combined with lemon oil and GA combined with peppermint oil generally produced the highest retention of nutritional constituents. (IQF) resulted in significantly higher carbohydrate, sugar, folic acid, inulin, cynarin, potassium, and iron contents than cooling or the combined chilling–freezing treatment. The beneficial effects of coatings were more pronounced under IQF conditions, indicating a synergistic interaction between storage temperature and coating formulation.

For polyethylene-packaged samples, GA + peppermint oil exhibited the highest retention of total sugars, folic acid, potassium, and iron, whereas (CMC) + clove oil resulted in the greatest accumulation of inulin and cynarin. Similar trends were observed under polypropylene packaging, although absolute values were consistently lower than those recorded in polyethylene packages.

### Changes in antioxidant compounds and bioactive constituents

Significant differences were observed among treatments regarding total phenolic content and vitamin C concentration (*P* < 0.0001) as Table 5. Edible coatings markedly increased phenolic accumulation compared with the control treatment. The highest total phenolic content was recorded in samples coated with GA + peppermint oil and CMC + clove oil, particularly under IQF storage.

Storage conditions also strongly influenced antioxidant retention. IQF-treated samples maintained significantly greater phenolic concentrations than chilled samples. Polyethylene packaging further enhanced the preservation of phenolic compounds compared with polypropylene packaging.

In contrast, vitamin C content declined in coated samples relative to controls, although the magnitude of the reduction depended on coating type and storage condition. Nevertheless, IQF storage consistently minimized vitamin C degradation compared with conventional cooling.

### Oxidative stress indicators and enzymatic browning

The oxidative status of fresh-cut artichoke was strongly affected by coating treatment and storage condition. Polyphenol oxidase (PPO) activity, malondialdehyde (MDA), hydrogen peroxide (H₂O₂), and superoxide radical contents were significantly reduced by edible coatings compared with the control (*P* < 0.0001) as Tables 6 and 7.

Among the coatings, GA + peppermint oil produced the greatest suppression of PPO activity and lipid peroxidation, indicating improved protection against enzymatic browning and oxidative deterioration. Similarly, coated samples exhibited markedly lower H₂O₂ and superoxide accumulation than untreated samples. IQF storage generated the lowest oxidative stress levels, whereas cooling alone resulted in the highest oxidative damage markers.

Polyethylene packaging further reduced oxidative stress parameters relative to polypropylene packaging, suggesting enhanced barrier properties and protection against oxygen-mediated deterioration.

### Texture preservation and weight loss

Texture retention was significantly influenced by coating formulation, storage regime, and packaging material (*P* < 0.0001) as Table 9. Coated samples maintained higher texture scores than controls throughout storage. GA + peppermint oil provided the greatest firmness retention, followed by whey protein + lemon oil.

Weight loss was substantially reduced by edible coatings. Whey protein + lemon oil showed the strongest ability to minimize moisture loss, while control samples exhibited the greatest weight reduction. Polyethylene packaging generally provided better moisture retention than polypropylene packaging, especially when combined with edible coatings.

### Ethylene production and respiration activity

Both ethylene evolution and respiration rate were significantly affected by storage conditions and coating treatments (*P* < 0.0001) as Table 10. Coated samples exhibited lower ethylene production and reduced respiration rates than untreated controls, indicating delayed metabolic activity and senescence.

The strongest reduction in ethylene release was observed in samples treated with GA + peppermint oil and CMC + clove oil. Likewise, IQF storage significantly suppressed respiratory activity compared with cooling. Polyethylene packaging further contributed to reduced metabolic activity, demonstrating its suitability for extending shelf life of fresh-cut artichoke.

### Microbiological quality

Microbial counts were significantly reduced by edible coatings and low-temperature storage. Under both polyethylene and polypropylene packaging systems, coated samples exhibited lower total bacterial counts and mold and yeast populations than the control treatment. In several coated samples stored under IQF conditions, molds and yeasts were not detected, confirming the effectiveness of the antimicrobial formulations as Tables 11 and 12.

The combined chilling–IQF treatment provided the greatest microbial suppression, particularly when coatings containing mint or clove essential oils were applied.

### Antimicrobial activity of edible films

The antimicrobial assay demonstrated clear inhibitory effects of edible films enriched with essential oils against foodborne microorganisms. Films containing GA + peppermint oil exhibited the largest inhibition zones against *Escherichia coli* and *Staphylococcus aureus*, whereas both formulations showed antifungal activity against *Aspergillus niger* as Table 13. These findings confirm the contribution of essential oils to the antimicrobial functionality of the developed coating systems.

### Mechanical and barrier properties of edible films

Mechanical characterization revealed significant variation among coating formulations. The GA + peppermint oil film exhibited the highest tensile strength, whereas whey protein + lemon oil showed the greatest elongation capacity as Table 14. CMC + clove oil generated the lowest water vapor transmission rate, indicating superior moisture barrier properties. These characteristics support the effectiveness of the developed edible films in preserving the quality of fresh-cut artichoke during refrigerated and frozen storage.

## Discussion

The present study demonstrates that combining edible coatings with low-temperature storage and appropriate packaging materials effectively preserved the nutritional, physiological, and microbiological quality of fresh-cut artichoke during two weeks of storage. The observed improvements can be attributed to the synergistic effects of coating-induced barrier properties, antioxidant activity of essential oils, and temperature-mediated reduction of metabolic processes^8,52,53^.

A major finding of this study was the enhanced retention of carbohydrates, proteins, sugars, inulin, cynarin, folic acid, and mineral elements in coated samples compared with the uncoated control. Fresh-cut processing disrupts cellular integrity and accelerates respiration, enzymatic degradation, and nutrient losses. The edible coatings likely acted as semi-permeable barriers that reduced gas exchange and moisture migration, thereby slowing metabolic consumption of stored reserves. Furthermore, the incorporation of essential oils may have stabilized cellular membranes and reduced oxidative degradation of sensitive compounds. Similar protective effects of edible coatings enriched with bioactive compounds have been reported for several minimally processed horticultural commodities^17,52,54^.

The observed increases in protein, inulin, cynarin, and total sugar contents during storage should not be interpreted as de novo biosynthesis of these compounds after harvest. Instead, these increases most likely reflect an apparent concentration effect resulting from gradual moisture loss and consequent reduction in tissue water content, which increases the relative concentration of dry matter constituents. Furthermore, the edible coatings acted as semipermeable barriers that reduced respiration, transpiration, and metabolic degradation processes, thereby improving the retention of nutritional and bioactive compounds throughout storage. Similar effects have been reported for coated fresh and fresh-cut horticultural products, where edible coatings reduced water loss, delayed senescence, and enhanced the preservation of quality-related metabolites^55–57^. In the case of soluble sugars, partial hydrolysis of reserve carbohydrates, particularly inulin, may also contribute to the increased sugar concentration. Therefore, the higher values recorded in coated samples are more appropriately attributed to enhanced preservation and retention of bioactive constituents rather than to their postharvest synthesis. The simultaneous increase in both inulin and soluble sugars may be explained by the dynamic equilibrium between inulin depolymerization and moisture loss during storage. Inulin is the principal storage fructan in artichoke tissues and may undergo partial hydrolysis, yielding fructooligosaccharides and fructose, thereby contributing to the accumulation of soluble sugars^58,59^. However, the protective effects of edible coatings and low-temperature storage likely minimized extensive carbohydrate degradation, allowing substantial retention of inulin while increasing the measurable concentration of soluble sugars on a fresh-weight basis. Similar behavior has been reported for fructan-rich horticultural products, where storage-induced modifications in fructan structure occurred simultaneously with enhanced retention of bioactive carbohydrates under optimized preservation conditions^60^.

Potassium and iron were selected as representative macro- and micronutrients because they are among the most nutritionally relevant mineral elements present in artichoke tissues. The objective of measuring these minerals was not to assess postharvest mineral biosynthesis, which does not occur after harvest, but rather to evaluate the ability of edible coatings and storage conditions to preserve mineral retention during storage. The relatively higher concentrations observed in coated samples may be attributed to reduced moisture loss, improved membrane integrity, and lower cellular leakage resulting from the protective barrier properties of the coatings. By limiting oxidative damage and preserving tissue structure, edible coatings likely minimized mineral losses and contributed to maintaining the nutritional quality of fresh-cut artichokes throughout storage, consistent with previous reports demonstrating that edible coatings reduce water loss and preserve cellular integrity in fresh horticultural products^53,61^.

The superior performance of (IQF) compared with conventional cooling can be explained by the rapid formation of small ice crystals, which minimizes structural damage and limits biochemical deterioration. Reduced enzymatic activity under freezing conditions likely contributed to the higher retention of sugars, inulin, and cynarin observed in the present study. The beneficial effect was particularly evident when IQF was combined with edible coatings, suggesting a synergistic interaction between physical and biochemical preservation mechanisms^10,62^.

Phenolic compounds constitute one of the most important antioxidant defense systems in artichoke tissues. The significant increase in total phenolic content in coated samples indicates that edible coatings were effective in protecting phenolic constituents from oxidation. Essential oils derived from mint, lemon, and clove are rich sources of phenolic and terpenoid compounds with strong antioxidant properties. These compounds may directly scavenge reactive oxygen species (ROS) and inhibit oxidation reactions occurring during storage. The enhanced phenolic preservation observed in polyethylene-packaged samples further highlights the importance of limiting oxygen penetration into packaged products^7,9,54,63^ .

The oxidative stress indicators measured in this study provide strong evidence supporting the protective role of edible coatings. Lower levels of hydrogen peroxide, superoxide radicals, and malondialdehyde were consistently observed in coated samples, indicating reduced accumulation of reactive oxygen species and lower membrane lipid peroxidation. Malondialdehyde is a widely recognized marker of oxidative membrane damage; therefore, its reduction suggests improved membrane integrity throughout storage. The simultaneous decline in ROS accumulation and lipid peroxidation demonstrates that the coatings effectively mitigated oxidative stress, which is one of the primary causes of quality deterioration in fresh-cut vegetables^64–66^.

Polyphenol oxidase (PPO) is a key enzyme involved in enzymatic browning and quality loss in fresh-cut produce. The substantial reduction in PPO activity observed in coated samples likely resulted from restricted oxygen availability at the tissue surface as well as the inhibitory effects of phenolic constituents present in the incorporated essential oils. Reduced PPO activity is particularly important for artichoke, which is highly susceptible to browning after tissue disruption. Consequently, the lower PPO activity observed in coated samples likely contributed to the improved appearance and quality retention during storage^8,67^.

Maintenance of texture is a critical determinant of consumer acceptance of fresh-cut products. The higher texture scores observed in coated treatments suggest that edible coatings reduced moisture loss and preserved cell wall integrity. The coatings likely formed a protective matrix that limited dehydration and reduced structural collapse of plant tissues. This interpretation is supported by the significantly lower weight loss recorded in coated samples. Reduced water loss not only preserves firmness but also limits concentration effects that accelerate physiological deterioration^22,68,69^.

Another important outcome of this work was the reduction in ethylene production and respiration rate. Fresh-cut processing typically stimulates wound-induced respiration and ethylene biosynthesis, accelerating senescence and quality deterioration. The lower respiratory activity observed in coated samples indicates that the coatings effectively modified the microenvironment surrounding the tissue and reduced oxygen availability for metabolic reactions. This reduction in metabolic intensity likely contributed to the improved retention of nutrients and antioxidant compounds observed throughout storage^12,20,70^.

Microbial growth is one of the primary factors limiting the shelf life of minimally processed vegetables, and the present results demonstrated a marked reduction in total bacterial and fungal populations in coated artichoke samples during storage. This reduction was consistent with the antimicrobial performance observed in the in vitro disc diffusion assay, where coating formulations exhibiting larger inhibition zones also showed greater effectiveness in suppressing microbial growth under refrigerated and frozen conditions. Such agreement between in vitro and in vivo results indicates that the antimicrobial constituents of essential oils retained their biological activity after incorporation into the coating matrices and application to the food surface, as previously reported for essential oil–based active coatings applied to fresh-cut fruits and vegetables^71–73^. Essential oils are known to exert antimicrobial effects through disruption of microbial cell membranes, increased permeability, and interference with essential metabolic processes, which collectively contribute to growth inhibition, a mechanism widely documented in recent reviews on plant-derived antimicrobials^7,9,74,75^.

The antimicrobial assay supported these observations by demonstrating inhibition of both Gram-positive and Gram-negative bacteria as well as fungal species. The strong inhibitory activity exhibited by coatings containing mint and lemon essential oils is consistent with the presence of terpenes, phenolics, and oxygenated volatile compounds known for their broad-spectrum antimicrobial properties. Therefore, the microbiological improvements observed during storage can be directly linked to the intrinsic antimicrobial activity of the coating formulations^62,63,77^.

The physicochemical properties of the developed films also contributed to their preservation efficiency. Variations in tensile strength, elongation, water vapor permeability, and transparency indicate that each coating matrix possessed distinct structural characteristics affecting its protective performance. Films exhibiting lower water vapor transmission rates provided more effective moisture barriers, while adequate mechanical strength ensured coating integrity during storage. These functional properties likely enhanced the ability of coatings to regulate gas exchange and maintain product quality^18,19,52^.

Overall, the results suggest that quality preservation in fresh-cut artichoke is governed by an integrated mechanism involving reduced oxidative stress, suppression of enzymatic browning, maintenance of membrane integrity, inhibition of microbial proliferation, and attenuation of respiratory metabolism^8,64,65^. Among the evaluated treatments, edible coatings enriched with essential oils, particularly GA +peppermint oil and whey protein+ lemon oil formulations, combined with IQF and polyethylene packaging, provided the most effective strategy for maintaining nutritional quality and extending the storage life of fresh-cut artichoke.

### Proposed mechanism of quality preservation

The applied coating matrices likely acted as semi-permeable barriers that restricted oxygen diffusion and regulated gas exchange between plant tissues and the surrounding environment. Reduced oxygen availability consequently limited the generation of reactive oxygen species (ROS), as evidenced by the lower accumulation of hydrogen peroxide (H₂O₂) and superoxide radicals observed in coated samples.

The attenuation of oxidative stress was associated with reduced lipid peroxidation and membrane deterioration, reflected by the significant decline in malondialdehyde (MDA) content. Improved membrane integrity likely contributed to the preservation of cellular compartmentalization and restricted the interaction between oxidative enzymes and their substrates. Consequently, polyphenol oxidase (PPO) activity was suppressed, reducing enzymatic browning and quality deterioration.

The reduction in oxidative damage further enhanced the retention of phenolic compounds, inulin, folic acid, vitamin C, and other nutritionally important constituents. Preservation of these bioactive molecules strengthened the endogenous antioxidant defense system of artichoke tissues, thereby creating a positive feedback mechanism that further limited ROS accumulation during storage. Simultaneously, reduced oxidative stress and improved membrane stability contributed to lower respiration rates and ethylene production, delaying senescence-associated metabolic processes. In parallel, the antimicrobial properties of the incorporated essential oils inhibited microbial proliferation, while the barrier characteristics of the coatings reduced moisture loss and maintained tissue turgor. Together, these physiological, biochemical, and microbiological responses resulted in enhanced texture retention, improved nutritional quality, and extended storage stability. Therefore, the preservation mechanism can be summarized as follows: reduced O₂ diffusion → lower ROS generation (H₂O₂ and O₂⁻) → lower PPO activity and MDA accumulation → improved retention of phenolics and nutritional compounds → reduced respiration and ethylene production → enhanced texture, microbial stability, and overall postharvest quality.

## Conclusion

The present study demonstrates that edible coatings based on whey protein, GA, and CMC, when enriched with natural essential oils, are highly effective in maintaining the postharvest quality of artichokes under different storage conditions. These coatings significantly reduced physiological deterioration, microbial growth, and oxidative damage compared with uncoated samples.

Among the tested formulations, GA and whey protein coatings showed the most pronounced effectiveness in preserving nutritional compounds and maintaining overall quality attributes during storage. The reduction in weight loss, respiration rate, ethylene production, and microbial load confirms the protective barrier function of the coatings, which limits oxygen exchange and inhibits microbial proliferation.

Additionally, the coatings contributed to the retention of bioactive compounds such as phenolics, ascorbic acid, inulin, and cynarin, thereby enhancing the functional quality of the stored artichokes. The observed decrease in oxidative stress markers and enzymatic browning further supports the role of edible coatings in delaying senescence and quality degradation.

In conclusion, edible coatings enriched with natural essential oils represent a promising, eco-friendly postharvest technology for extending the shelf life and improving the quality of fresh artichokes. Future studies should focus on scaling up the application and evaluating consumer acceptance and industrial feasibility.

## Supplementary Information

Below is the link to the electronic supplementary material.

Supplementary Material 1

## Data Availability

The datasets generated during and/or analysed during the current study are available from the corresponding author on reasonable request.

## Declarations

### Permission statement

Permission has been granted by Al-Rayan Artichoke Farm and Factory to collect samples for the research study on April 20 in the year 2024 and 2025.
