# Supplementary material for: Evaluation of edible coating on quality, biochemical components and microbial community under cooling and freezing conditions on fresh-cut artichoke
Source: Sci Rep. 2026 Aug 8;16:24537. doi: 10.1038/s41598-026-63629-2 (PMC13452818; doi:10.1038/s41598-026-63629-2)
Supplement: Supplementary file 1 [file 41598_2026_63629_MOESM1_ESM.docx]

***Staphylooccus aureus at* Arabic gum +mint oil**

***Staphylooccus aureu****s* **at whey protein +limon oil**

**(A) *E.Coli***

**(B) *E.Coli***

(A**) *E.Coli at* Arabic gum +mint oil**

**(B) *E.Coli* at whey protein +limon oil**

(A) ***B.cereus	at* Arabic gum +mint oil**

**(A) *Aspergillus niger***

**(B)*Aspergillus niger* at whey protein +limon oil.**
